# Dietary practices in isolated First Nations communities of northern Canada: combined isotopic and lipid markers provide a good qualitative assessment of store-bought vs locally harvested foods consumption

**DOI:** 10.1038/nutd.2013.34

**Published:** 2013-10-21

**Authors:** T Seabert, S Pal, E M Krümmel, J M Blais, P Imbeault, M A Robidoux, F Haman

**Affiliations:** 1 Program for Chemical and Environmental Toxicology, Department of Biology, 30 Marie Curie Drive, University of Ottawa, Ottawa, Ontario, Canada; 2 School of Human Kinetics, 125 University St, University of Ottawa, Ottawa, Ontario, Canada; 3 Indigenous Health Research Group, 125 University St, University of Ottawa, Ottawa, Ontario, Canada

**Keywords:** aboriginal, lipids, isotopic tracers, dietary practices

## Abstract

**OBJECTIVE:**

In First Nations communities of northwestern Ontario, where rates of type 2 diabetes mellitus are some of the highest in the world, ascertaining wild food dietary practices is extremely challenging owing to seasonal availability, environmental factors, life circumstances and language/cultural barriers. The purpose of this study was to determine whether analysis of isotopic and fatty acid (FA) profiles could provide more comprehensive information to discriminate between three categories of wild food consumption (that is, plants and animals) in two isolated First Nations communities of northwestern Ontario. In addition, this analysis also highlights whether wild food consumption as practiced in these two communities can increase circulating levels of polyunsaturated FAs (PUFAs), which provide a number of important metabolic benefits that could impact the prevention/treatment of T2DM.

**RESULTS:**

^13^C enrichment (in expired CO_2_, plasma and hair), ^15^N enrichment (in hair) and FA profiles in plasma phospholipids (phospholipid fatty acid (PL-FA)) were quantified in men and in women consuming various amounts of wild food. ^13^C/^12^C ratios were lower and ^15^N/^14^N ratios were higher in participants consuming wild food at least once a week. In addition, FA results indicated that the relative contributions of 20:4 Ω-6 and 22:6 Ω-3 to total PL-FAs were higher and 18:2 Ω-6 lower in wild food consumers.

**CONCLUSION:**

Together, these findings confirm that isotopic and lipid markers discriminate between the different wild food categories in these two First Nations communities. Knowing the close relationship between dietary intake and the potential role of PUFA in the prevention/treatment of obesity and obesity-related diseases, it is critical to accurately measure the composition of diet for individuals in their specific environments.

## Introduction

In research and clinical perspectives, adequate assessments of dietary practices are essential for the prevention/treatment of type 2 diabetes mellitus. This holds particularly true in Canada's First Nations communities of northwestern Ontario where rates of type 2 diabetes mellitus are some of the highest in the world.^1, 2, 3, 4, 5^ Although store-bought foods are available in these isolated communities, they are generally of poor quality and expensive.^6^ Some nutritious choices are available for purchase but they are generally five to six times more expensive than in southern Canadian cities. In this context, locally harvested foods remain a key source of nourishment for these communities, making its incorporation into diet an important strategy for reducing high rates of obesity and type 2 diabetes mellitus. Locally harvested plants and animals are significant sources of dietary protein, essential minerals, vitamins and polyunsaturated fatty acids (PUFAs),^7, 8, 9^ particularly long-chain Ω-3 fatty acids (FAs). These Ω-3 FAs have protective benefits against cardiovascular disease, obesity and diabetes^10, 11^ which continue to be much more prevalent in aboriginal populations.^1, 12, 13^ The environment around remote communities of northwestern Ontario provides access to a wide range of fish, mammals, birds and wild berries that can be consumed throughout the year.^14^ However, these wild foods are far from being accessible in sufficient amount to all community members. Ascertaining the extent to which these foods are consumed is challenging owing to their seasonal availability, environmental factors and life circumstances that impact access to land-based foods. This unique context makes accurate and objective assessments of overall food consumption difficult, which is exacerbated by language/cultural barriers and reporting bias.

Methods to assess dietary intake using isotopic and lipid markers may assist in mitigating challenges with communication and reporting. It is well established that isotopic enrichment and FA profiles of consumed foods are reflected in the organism and can be quantified in blood, expired gases and tissue biopsies.^15, 16, 17, 18, 19^ For example, some studies have used stable isotope ratios of carbon (^13^C/^12^C) and nitrogen (^15^N/^14^N) to estimate the relative consumption of sugars and sweeteners made from corn and sugar cane^20^ and the consumption of specific types of proteins between groups of individuals.^15, 17, 18, 20, 21^ Similarly, intervention studies in humans and in other animals showed that dietary FAs are reflected in the FA profile of membrane phospholipids (PLs).^22, 23, 24, 25^ Traditionally, the hunter/gatherer diet north of the 49th parallel was rich in proteins (∼35% of total energy intake (E)), rich in lipids (∼43% E), and low in carbohydrates (CHO; ∼22% E).^26^ The CHO consumed were mainly from C_3_ photosynthesis plants low in carbon-13 as sugars from corn and sugar cane were not available.^26, 27, 28^ Proteins and lipids were obtained from hunting and fishing and constituted at least 70% of their subsistence.^26^ Proteins from wild meats, especially fish, are naturally enriched in ^15^N (∼14 *δ*^15^N‰) compared with market meats (∼4 *δ*^15^N‰).^21^ Moreover, high wild meat consumption provides a greater diversity of FA intake.^9^ For example, PUFA intake of hunter–gatherer societies was estimated at 27 g·day^−1^ with 42% of linoleic acid, 41% of alpha-linolenic acid, 11% of 20–22C Ω-6 FAs and 6% of 20–22C Ω-3 FAs for a Ω-3 PUFA balance of 47%.^29^ Whether the combined measurements of isotopic enrichment (^13^C and ^15^N) and FA profiles would allow researchers to discriminate between dietary practices in First Nations communities of Northwestern Ontario remains to be determined.

The purpose of this study was to quantify differences in the quality of CHO, lipid and protein consumed in two Oji-Cree communities of northwestern Ontario. More specifically, ^13^C enrichment (in expired CO_2_, plasma and hair), ^15^N enrichment (in hair) and FA profiles in plasma PLs were quantified in men and in women consuming various amounts of wild food. Based on the combined results obtained by a number of researchers,^15, 17, 18, 20, 21, 22, 23, 24, 25, 30^ we anticipated that wild food consumers would show lower ^13^C enrichment in all compartments measured, higher ^15^N enrichment and higher PUFA levels in plasma PL.

## Methods

### Participants

This study was conducted according to the guidelines in the Declaration of Helsinki and all procedures involving human subjects/patients were approved by the University of Ottawa Research Ethics Board and Health Canada Ethics Board. Written informed consent was obtained from all volunteers. The study was conducted in Wapekeka (Angling Lake) and Kasabonika Lake First Nations in northwestern Ontario, Canada. Inclusion criteria required that individuals be Aboriginal, over 18 years, non-pregnant and free of type 1 diabetes. The number of individuals participating in each community (39 in Wapekeka and 32 in Kasabonika) represents ∼9% of the eligible population in Kasabonika and 24% of the eligible population in Wapekeka at the time of the study. In order to potentiate differences in dietary behaviour between individuals, data collection was performed over a period of 2 months when wild food consumption was known to be high in these communities (late September to early November 2007). Participants were recruited using a mixed-method ethnological approach combining semi-structured interviews, food frequency questionnaires, 3-day dietary records and 24-h dietary recalls. As presented in Table 1, the 71 participants who participated in this study were separated into three groups according to the estimated frequency of wild food consumption: once a week or more (WF3), between once a week and once a month (WF2) and once a month or less (WF1).

### Sample collection

Volunteers were asked to report at the nursing station (Kasabonika) and clinic (Wapekeka) located in each community at ∼0900 following 24 h without strenuous excercise and a 12 h fast. First, height and weight were measured and used to calculate body mass index (BMI) by dividing body weight (in kilograms, kg) by square height (in metres, m). For the collection of expired CO_2_, participants exhaled in a 10 ml vacutainer tube containing no additives. Tubes were sealed with a rubberized cap and secured in place with paraffin tape for transport to the University of Ottawa (Ottawa, Ontario, Canada). Blood samples were taken from the antecubital vein (left arm) in 4 ml vacutainers containing an anticoagulant (ethylenediaminetetraacetic acid). Upon collection, blood samples were immediately placed on ice and centrifuged at 3500 g. Plasma and red blood cells were separated and frozen at −20 °C. At the end of the study, samples were transported frozen to a −80 °C freezer at University of Ottawa (Ottawa, Ontario) until analysis. Hair samples were collected from the nape of the neck as near as possible to the scalp using stainless steel scissors, and were sealed separately in labelled bags for safe transportation to the University of Ottawa. Hair was then cleaned by soaking in a 2:1 chloroform:methanol solution to remove any residues and then rinsed several times with distilled water. Samples were thoroughly dried before performing any analysis.

### Sample analysis

^*13*^*C/*^*12*^*C ratio in expired CO*_*2*_. ^13^C enrichment in expired CO_2_ was measured at the GEOTOP UQAM/McGill Stable Isotope Laboratory (Université du Québec à Montréal, Montréal, Canada) in a Prism mass spectrometer (VG, Manchester, UK). *In plasma glucose.* 1 ml of plasma was deproteinized, and the glucose was then separated by double-bed ion exchange chromatography by running the supernatants through superimposed columns (resins: AG 50 W-X8 H+, 200–400 mesh and AG 1-X8 chloride, 200–400 mesh), as previously described by Peronnet *et al.*^31^ Following evaporation, glucose was combusted (60 min at 400 °C) in the presence of copper oxide. CO_2_ was recovered and measured at the GEOTOP UQAM/McGill Stable Isotope Laboratory (Université du Québec à Montréal, Montréal, Canada) in a Prism mass spectrometer (VG, Manchester, UK). *Isotope ratios in hair*. ^13^C and ^15^N enrichment in hair was measured at the G.G. Hatch Stable Isotope Laboratory, University of Ottawa. Cleaned hair samples (∼0.6 milligram) were cut, weighed and placed into tin capsules (8 × 5 millimetres (mm), Isomass Scientific (Calgary, AB, Canada). Isotopic enrichment was determined by the analysis of CO_2_ and N_2_ produced by flash combustion at 1800 °C on a CE 1110 Elemental Analyzer followed by gas chromatographic separation and on-line analysis by continuous-flow with a DeltaPlus Advantage isotope ratio mass spectrometer coupled with a ConFlo interface. Data were normalized using internal standards previously calibrated with International standards IAEA-CH-6, IAEA-NBS22, IAEA N1, IAEA-N2, USGS-40 and USGS-41.

Isotopic composition in hair samples, in expired CO_2_ and in CO_2_ obtained from plasma glucose was expressed as *δ* values (‰) compared with the V-PDB standard for ^13^C and atmospheric N_2_ for ^14^N, respectively:^32, 33^


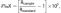


where X is ^13^C and ^15^N and R is the corresponding ratio of ^13^C/^12^C and ^15^N/^14^N in the sample and standard, respectively.

#### FA profiles of plasma PL

Circulating lipids were extracted twice in chloroform–methanol (2:1 v/v) from 200 μl of plasma collected in the fasting state according to the Folch method.^34^ Extracted lipids were then suspended in chloroform in which PLs were separated by filtration on Superclean solid-phase extraction tubes (1 ml LC-NH_2_; Sigma-Aldrich, St Louis, MO, USA) as previously described^35^ and then eluted with methanol. Two hundred micro liters of margaric acid (17:0; 30 μg per 100 μl hexane) was added as an internal standard and PLs were then trans-esterified with acetyl chloride in methanol for 2 h at 90 °C. After evaporation, the newly formed FAs methyl ester were dissolved in 60 μl isooctane and 2 μl were injected in a Hewlett-Packard gas chromatograph (HP 9890 with HP 7683B autosampler) equipped with a flame-ionization detector and a 60 m fused silica column (DB-23; J&W Scientific, Folsom, CA, USA). The carrier gas was nitrogen and detector gases were hydrogen and air. Injection port temperature was set at 220 °C and the detector at 240 °C. The temperature was 185 °C for 35 min after injection, raised to 210 °C at a rate of 5 °C min^−1^, and kept at 210 °C for an additional 10 min. Exact retention times of each FA were determined with pure standards (Sigma-Aldrich). Plasma PL concentrations were measured by colorimetry using the PL-C microtiter procedures kit (Wako Diagnostics, Chemicals, Richmond, VA, USA). Because samples were not store in transported in N_2_ filled vacutainers, it is possible that some hydrolysis of PL occurred resulting in a release of FAs. These FAs would not have been included in the PL fraction following the filtration through the solid-phase extraction tubes (described above). However, it was assumed that the hydrolysed PL would release FAs in the same proportion as what remained in the non-hydrolysed PL fraction. FA profiles were presented as per cent contribution of individual FA to total plasma PL-FA. Only FAs accounting for >5% of total PL-FA were presented in this analysis. Degree of unsaturation of PL-FA was calculated using the following formula:^36^


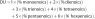


### Statistical analysis

Data were expressed as mean+s.e. A one-way ANOVA with between-subjects design was used to analyse differences in ^13^C/^12^C ratios, ^15^N/^14^N ratios and relative contributions of individual FAs to total PL-FA. Bonferonni's multiple comparisons *post hoc* test was used, where applicable. A two-tailed *P*-value of <0.05 was considered significant. All analyses were performed using SPSS for Windows (version 16.0; SPSS Inc., Chicago, IL, USA) or GraphPad Prism version 5.00 for Windows (GraphPad, San Diego, CA, USA). All parameters were normally distributed (according to the Shapiro–Wilk test) and the homogeneity of variance between our groups was equal, based on the Levene's test. Despite limited sample size, the effect size for the differences observed between our groups was large (that is, *d*>0.8). Because 25 of the 71 participants suffered from type 2 diabetes mellitus, we conducted a chi-square test and Pearson correlations between HOMA-IR to determine whether this chronic disease could influence the results of this study. We found that proportion of type 2 diabetic individuals did not differ between food groups (chi-square=2.72, *P=*0.26). In addition, isotopic and FA markers did not correlate with HOMA-IR scores (*P=*0.47–0.98). Based on these two results, we confirm that our findings remain the same regardless of whether type 2 diabetic individuals are included in the analyses.

## Results

### Participants

Differences in wild food categories, age, sex ratios, weight and body mass index between individuals and within each food behaviour group are presented in Table 1. Even though no difference in body weight (*P=*0.13) and body mass index (*P=*0.10) were found between groups, WF1 were 1.8 years and 12.1 years younger than WF2 (*P=*0.02) and WF3 (*P=*0.02), respectively.

### Isotopic composition

**Figure 1:**
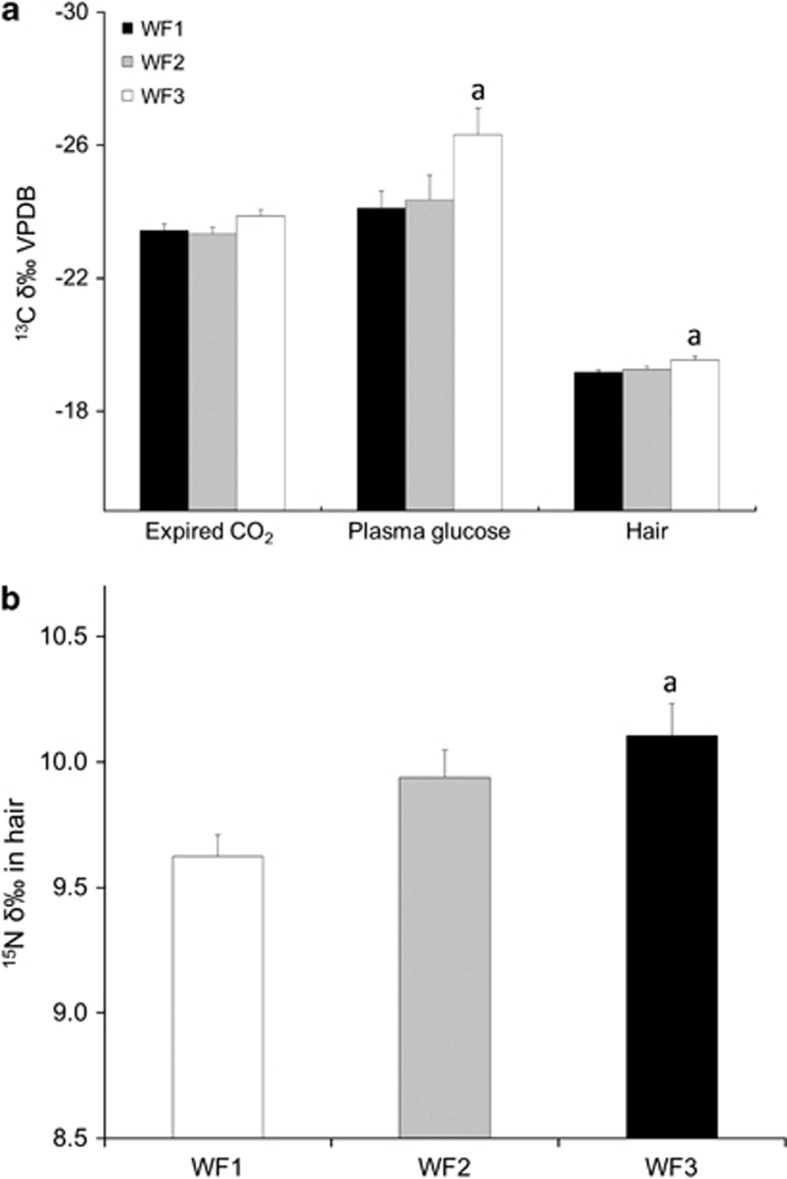
(**a**) *δ*^13^C±s.e. (‰, hair, expired CO_2_ and plasma) and (**b**) hair *δ*^15^N±WFSE (‰) for WF1, WF2 and WF3 food consumption groups. Values are means±s.e. a, significantly different from WF1.

Differences in ^13^C/^12^C ratios for expired CO_2_, plasma glucose and hair for WF1, WF2 and WF3 are presented in Figure 1a. No differences in the ^13^C/^12^C ratio was found in expired CO_2_ with *δ*^13^C values averaging −23.4±0.2‰ for WF1, −23.3±0.2‰ for WF2 and −23.9±0.2‰ for WF3 (*P=*0.16). In contrast, the average plasma glucose ^13^C/^12^C ratio was lower in WF1 (*δ*^13^C of −24.1±0.5‰) than in WF3 (*δ*^13^C of −26.3±0.8‰ *P=*0.05). Values in WF2 for this compartment (*δ*^13^C of −24.3±0.8‰) were not different from WF1 (*P=*0.16) and WF3 (*P=*1.00). Similar results were found for ^13^C/^12^C ratios in hair where WF1 displayed a lower average (*δ*^13^C of −19.2±0.1‰) than in WF3 (*δ*^13^C of −19.5±0.1‰ *P=*0.019). No differences were observed between WF2 (*δ*^13^C of −19.2±0.1‰) and WF1 (*P=*0.13) and between WF2 and WF3 (*P=*1.00).

Differences in ^15^N/^14^N ratios found in hair in WF1, WF2 and WF3 are presented in Figure 1b. As for ^13^C/^12^C ratios in plasma glucose and hair, a significant difference was observed for the ^15^N/^14^N ratio in WF1 (*δ*^15^N of 9.6±0.1‰) compared with WF3 (*δ*^15^N of 10.1±0.1‰) (*P=*0.006). Values in WF2 (*δ*^15^N of 9.9±0.1‰) were not different from WF1 (*P=*0.90) or WF3 (*P=*0.116).

### FA profiles of plasma PLs

**Figure 2:**
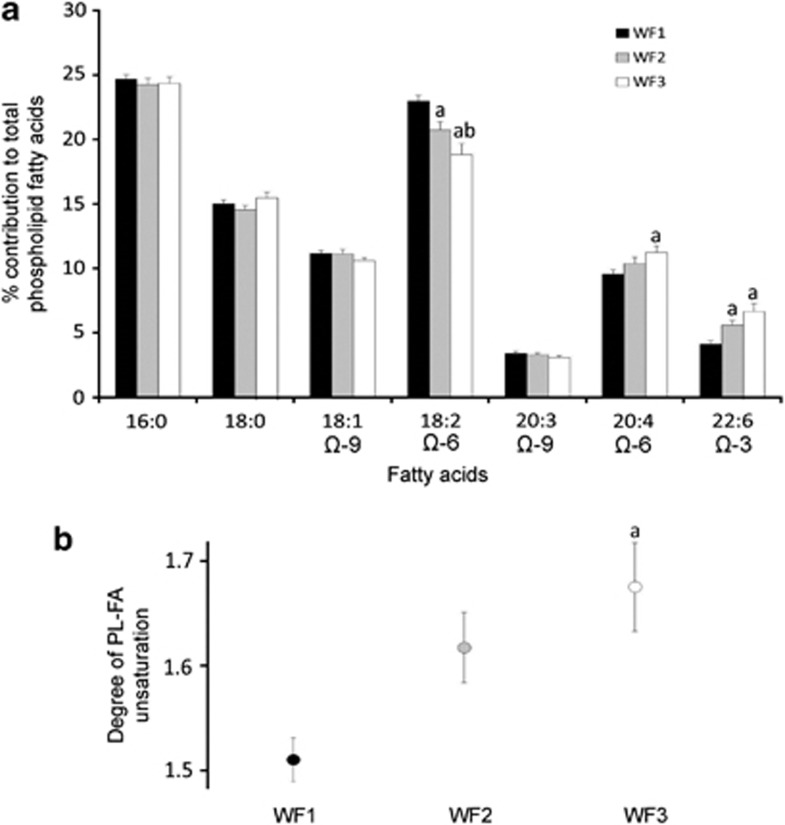
(**a**) Per cent contribution of individual fatty acids to total phospholipids (%PL-FA) and (**b**) degree of unsaturation (DU) of PL-FA measured in WF1, WF2 and WF3 food consumption groups. Only fatty acids accounting for >2% of total PL-FA are presented. Values are means±s.e. a, significantly different from WF1; and b, significantly different from WF2.

Differences in the FAs composition and degree of PL-FA unsaturation for WF1, WF2 and WF3 are shown in Figure 2. There were no differences in the per cent contribution of 16:0 (*P=*0.76), 18:0 (*P=*0.18), 18:1 Ω-9 (*P=*0.22) and 20:3 Ω-9 (*P=*0.34) to total PL-FA between WF1 (24.7±0.3%, 15.0±0.3%, 11.2±0.2% and 3.4±0.2%, respectively), WF2 (24.2±0.5%, 14.5±0.3%, 11.1±0.3% and 3.3±0.2%, respectively) and WF3 (24.3±0.5%, 15.5±0.4%, 10.6±0.2% and 3.0±0.2%, respectively) (Figure 2a). The relative contribution of 18:2 Ω-6 to total PL-FA decreased progressively with increasing level of wild food consumption from 23.0±0.5% in WF1 to 20.7±0.7% in WF2 to 18.8±0.9% in WF3 (*P<*0.001). Conversely, per cent contribution of 20:4 Ω-6 to total PL-FA increased overall from WF1 (9.6±0.3%) to WF3 (11.2±0.5%) (*P=*0.03). No difference in this FA was found between WF2 (10.4±0.5%) and WF1 (*P=*0.60) or WF3 (*P=*0.57). Finally, per cent contribution of 22:6 Ω-3 to total PL-FA was lower in WF1 (4.1±0.2%) when compared with WF3 (6.6±0.6%) (*P<*0.0001). However, no difference for 22:6 Ω-3 was observed between WF2 (5.6±0.4%) and WF1 (*P=*0.27) although this FA was lower in WF2 than in WF3 (*P=*0.04). As shown in Figure 2b, degree of unsaturation of PL-FA increased from WF1 (1.51±0.02%) to WF3 (1.68±0.04%) (*P=*0.001). No difference in degree of unsaturation was found between WF2 (1.62±0.03%) and WF1 (*P=*0.38) or WF3 (*P=*0.12).

**Figure 3:**
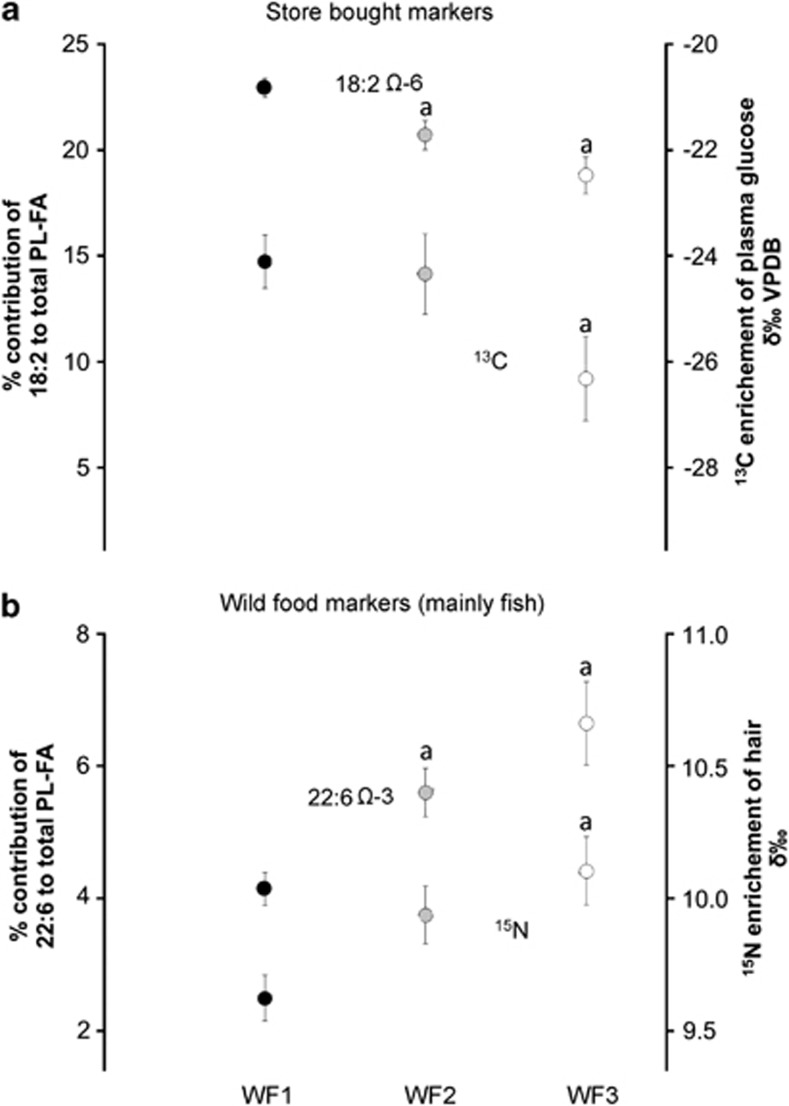
Isotopic and fatty acid markers of (**a**) store-bought and (**b**) wild food consumption (focus on fish). Values are means±s.e. a, significantly different from WF1.

Figure 3 summarizes and illustrates findings for isotopes ratios and FA profiles of plasma PLs as they relate to store-bought markers (^13^C/^12^C and 18:2 Ω-6) and fish consumption markers (^15^N/^14^N and 22:6 Ω-3). For store-bought food markers, ^13^C/^12^C ratios and the relative contribution of 18:2 Ω-6 to total PL-FA decreased overall from WF1 to WF3 (*P=*0.05 and *P<*0.0001, respectively). For fish consumption markers, ^15^N/^14^N ratios and the relative contribution of 22:6 Ω-3 to total PL-FA increased overall from WF1 to WF3 (*P=*0.006 and *P<*0.0001, respectively). In addition, ^13^C/^12^C ratios and the relative contribution of 18:2 Ω-6 to total PL-FA as well as ^15^N/^14^N ratios and the relative contribution of 22:6 Ω-3 to total PL-FA were significantly correlated (*r=*0.51, *P<*0.0001 and *r=*0.58, *P<*0.05, respectively).

## Discussion

The purpose of this study was to determine whether analysis of isotopic and FA profiles could provide more comprehensive information to discriminate between various levels of wild food consumption in isolated First Nations communities of northwestern Ontario. As anticipated, isotopic results showed that ^13^C/^12^C ratios were lower and ^15^N/^14^N higher in participants consuming wild food at least once a week (Figure 1). In addition, FA results indicated that the relative contribution of 20:4 Ω-6 and 22:6 Ω-3 to total PL-FA was higher and 18:2 Ω-6 lower in wild food consumers (Figure 2). Together, these findings confirm that isotopic and lipid markers discriminate between the different wild food groupings in these two First Nations communities (Figures 1, 2, 3). Such an assessment is important to determine the relationship between dietary practices and the development/treatment of chronic diseases.

### Isotopic dietary markers

The traditional First Nations diet continues to be undermined by the access to low quality market foods in northern Canadian First Nations communities.^37^ The calorie dense, low quality market foods that are presently available in the two First Nations communities depicted in this study are rich in CHO derived from corn that contains a high natural abundance of ^13^C (*δ*^13^C of −11‰).^20, 38^ In contrast, traditional hunter–gatherer diet consists of plants relying on C_3_-photosynthesis with a lower natural abundance of ^13^C (*δ*^13^C of −25‰). Based on these important differences in ^13^C enrichment, many have advocated the value of carbon isotope ratios (^13^C/^12^C) as a means to differentiate dietary preferences/behaviours.^15, 17, 18, 20^ In our study, results indicated that differences in the quality of CHO consumed between participants were sufficient to obtain significant differences in average ^13^C/^12^C ratios in blood or hair between low- (WF1; less than once a month) and high-wild food consumers (WF3; more than once a week). However, ^13^C enrichment was not sensitive enough to discriminate between the quality of CHO consumed in WF1 and WF3 from the intermediate wild food consumers (WF2; less than once a week but more than once a month). In addition, contrary to what was anticipated, breath CO_2_ did not allow us to differentiate the three levels of wild food consumption. This might not be that surprising as ^13^CO_2_ abundance in breath CO_2_ is not only influenced by diet but also by the ^13^C abundance of the substrate being oxidized.^38, 39^ As fuel selection was not quantified in this study, it is difficult to speculate on whether the lack of differences in ^13^C/^12^C between groups is due to differences in substrate utilization.^39^ Nevertheless, findings from this study show that ^13^C/^12^C ratios in blood glucose and in hair are the most reflective of dietary practices in these communities.

In addition to ^13^C/^12^C ratios, nitrogen isotope ratios (^15^N/^14^N) have proven to be a powerful dietary marker for wild food and fish consumption in this study. This outcome corroborates several other studies that have shown hair *δ*^15^N to track animal protein consumption among vegans, vegetarians and omnivores.^15, 17, 18^ Compared with vegans and vegetarians, omnivores are feeding at the highest trophic level and thus have a greater enrichment of *δ*^15^N in their hair due to higher animal protein intake. As expected, in our study, individuals consuming greater amounts of wild fish and hunted meats display higher hair *δ*^15^N values. More specifically, community members that consumed wild meat more than once a week (WF3) had higher *δ*^15^N values than participants who consumed wild meats less than once a month (WF1). However, as for ^13^C/^12^C ratios in blood and hair, variations in diet were not sufficient to find differences between WF2 and the two other groups. It is important to note that higher *δ*^15^N values observed in the WF3 are likely attributed to the high consumption of wild fish. In these two communities, wild fish is a staple food available in large amounts most of the year.

### FA dietary markers

In addition to higher levels of ^15^N isotope, wild foods are particularly rich in 20–22C Ω-3 FAs.^9, 21, 40, 41^ Previous work has shown that PL-FA were highly reflective of FA intake for 3–6 weeks prior to the blood draw.^42, 43^ This is mainly due to the fact that proportion of PUFA in plasma PL is tightly regulated and approximate 40% of total FA.^44^ Here, results showed that 22:6 Ω-3 (6.6±0.6% PL-FA) and 20:4 Ω-6 (11.2±0.5% PL-FA) were, respectively, 1.6-times and 1.2-times higher, whereas 18:2 Ω-6 was 1.2-times lower (18.8±0.9% PL-FA) in WF3 when compared with WF1 (4.1±0.2, 9.6±0.3, 23.0±0.5% PL-FA, respectively). In the intermediate WF2, measurements of PL-FA were sensitive enough to discriminate the relative contribution of 18:2 Ω-6 from that of the two extreme groups. For 22:6 Ω-3, values for WF2 were different from those found for WF3 (Figure 2a). In addition, essential FA and degree of unsaturation were, respectively, 1.2- and 1.1-times higher between WF3 (23.4±1.1% PL-FA and 1.68±0.04) and WF1 (18.9±0.6% PL-FA and 1.51±0.02). Together, these results indicate that measurements of PL-FA composition reflect the dietary practices of community members, they also suggest strongly the provenance of foods consumed (that is, wild food or store-bought).

### Wild food vs store-bought

When compared with westernized foods obtained in stores and restaurants, adherence to land based foods is often recognized to provide several health benefits for indigenous populations.^1, 2, 45, 46, 47, 48^ Figure 3 summarizes key results from the presented study as they relate markers of store-bought vs wild food consumption. Community *store-bought food* is highly processed and is particularly rich in linoleic acid (18:2 Ω-6) derived from soybean oil and rich in refined sugar derived from corn and sugar cane containing high level of ^13^C label.^20, 21, 49^ In contrast, *wild foods* have higher 20–22C Ω-3 FAs, much lower linoleic acid and lower α-linolenic acid^29^ and contain virtually no refined sugars (*δ*^13^C∼−25‰). In addition, proteins from wild meats, especially fish, are naturally enriched in ^15^N (*δ*^15^N ∼14‰) compared with market meats (*δ*^15^N ∼4‰).^21^ Although historical diet was entirely land based, most individuals living in remote First Nations communities today have to rely on store-bought foods to sustain their dietary needs and complement wild food consumption. Interestingly, the parallel increase in 22:6 Ω-3 and *δ*^15^N of values strongly suggest that fish is a key source of meat for wild food consumers in these communities; a finding confirmed by the information obtained during the interviews. Clearly, our study showed that isotopes and FA measurements can be used to establish significant differences in the relative contribution of store-bought vs wild food consumption in First Nations communities. However, it seems that this is only possible when extremes in wild food consumption are considered; that is, wild food more than once a week or wild food less than once a month. Results show that only 22:6 Ω-3 and *δ*^13^C can be differentiated between intermediate WF2 and high wild food consumers in WF3. This finding exposes the importance of carefully recruiting participants in research attempting to quantify the potentially beneficial effects of wild food consumption on rates of chronic diseases. Typically, studies interested in estimating dietary pattern within a population would use epidemiological methodologies involving excessively large cohorts (ex: >10 000 samples) and food frequency questionnaires.^50, 51^ However, this approach is difficult in remote, isolated First Nations communities where available cohorts are small and where it is difficult to assess precisely the dependence on locally harvested/hunted foods.^46^ In this context, many researchers interested in assessing dietary intake rely on self-reporting by participants which has obvious limitations. Dietary assessment tools such as individual interviews, food frequency questionnaires and dietary recalls are essential to understanding dietary behaviours; however, they can be problematic owing to, among other things, under-reporting,^30^ individual privacy, bias, cost, and time required of both participants and researchers.^21, 52, 53, 54, 55^ Although dietary marker-based methods provide less qualitative information, they are relatively free of any bias^56^ and can help to validate dietary surveys by identifying inconsistencies between tissue concentrations and dietary intake.

## Conclusion

Stable isotopes and FA profiles as dietary markers have been used widely in both human and animal dietary studies, however none have successfully combined these methods to assess well-defined dietary behaviours in northern Canadian First Nations communities. With large differences in dietary behaviours observed within First Nations communities, analysis of stable isotopes and FA profiles of PLs can provide quantitative information about an individual's diet and can contribute greatly to Aboriginal health and nutrition studies by providing a ‘non-invasive, simple, yet powerful tool for monitoring dietary pattern'.^21^ The use of these methods can be directly applied to assessing dietary behaviours within and between First Nations communities. Knowing the close relationship between dietary intake and the development/prevention of obesity and obesity-related diseases, it is critical to accurately measure the composition of diet for individuals in their specific environments. This information will be key for the development of region specific chronic disease prevention strategies.

## Figures and Tables

**Table 1:** Characterization of participants for wild food categories WF1 (⩽ once a month), WF2 (once a week>WF2>once a month) and WF3 (⩾ once a week) dietary categories

	*WF1* n*=28 (13*♂*,15*♀)	*WF2* n*=22 (6*♂*,16*♀)	*WF3* n*=21 (12*♂*,9*♀)
Age (y)	41±2	41±2	51±4 ya,^b^
Weight (kg)	89±3	85±3	95±3
BMI (kg m^−2^)	32±1	31±1	34±1

Abbreviation: BMI, body mass index.

a Significantly different from WF1.

b Significantly different from WF2.
